# Exploring the acceptability of engaging in physical activity amongst older adults living in socioeconomically deprived areas after the COVID-19 pandemic: a qualitative study

**DOI:** 10.1186/s12889-025-24981-6

**Published:** 2025-10-29

**Authors:** Danielle Harris, Ashley Gluchowski, Alex Hall, Emma Elliott, Luke Munford

**Affiliations:** 1 School of Health Sciences, Faculty of Biology, Medicine & Health, The University of Manchester, Manchester, M13 9PL UK; 2 School of Health & Society, University of Salford, Salford, M6 6PU UK; 3 National Institute for Health and Care Research, Applied Research Collaboration-Greater Manchester, School of Health Sciences, Faculty of Biology, Medicine and Health, The University of Manchester, Manchester, M13 9PL UK

**Keywords:** Physical activity, Older adults, Lower socioeconomic status, Socioeconomic deprivation, COVID-19 pandemic

## Abstract

**Background:**

Older adults from lower socioeconomic status (SES) groups have lower physical activity levels compared to those from higher SES groups. During the COVID-19 pandemic, these disparities in physical activity persisted, or even widened in the UK and other high-income countries. This qualitative study investigated the views of older adults residing in areas of high socioeconomic deprivation, around the acceptability of engaging in physical activity in the aftermath of the COVID-19 pandemic.

**Methods:**

Semi-structured interviews with 16 older adults living in areas with high levels of socioeconomic deprivation in Manchester, UK were conducted. Most participants (*n* = 12) were not meeting physical activity guidelines. An inductive reflexive thematic analysis was undertaken.

**Results:**

Six themes were identified which related to the acceptability of engaging in physical activity amongst this group: (1) The COVID-19 pandemic - a teachable moment for physical activity, (2) Physical activity - for purpose or pleasure?, (3) Focus on what you can do rather than what you can’t do, (4) Opportunities to be physically active (which consisted of two sub-themes: Place and Time), (5) Importance of social environment, and (6) Physical activity messaging and perceptions.

**Conclusions:**

An increased motivation to do more physical activity post-pandemic was identified amongst this population, suggesting now is a good time to act for intervention development to capitalise on this. Better messaging is needed to communicate and improve the accessibility of physical activity guidelines amongst this population. To increase engagement in leisure-time physical activity amongst this population, future interventions and messaging should emphasise fun and enjoyment.

**Supplementary Information:**

The online version contains supplementary material available at 10.1186/s12889-025-24981-6.

## Background

Physical activity provides many health benefits for older adults, such as reduced risk of cardiovascular diseases [1, 2], prevention of frailty [3], reduced risk of falls [4], improvements in cognitive function [5], better levels of mental health [6], higher quality of life [7], as well as reduced mortality [8, 9]. There are different age thresholds according to different definitions of the term ‘older adult’ dependent on organisations and/or countries, with the United Nations typically using 60 years and over [10]. However, with regards to physical activity guidelines the World Health Organization (WHO) categorises older adults as those aged 65 years or older [11]. For the purposes of this paper, when referring to older adults this applies to those aged 65 years or older, unless otherwise specified. The WHO recommends that older adults perform at least 150–300 min of moderate-intensity aerobic activity, or 75–150 min of vigorous-intensity aerobic activity per week, as well as functional strength and balance training on at least three days per week [11]. However, physical activity levels decline with increasing age [12], and nearly half (44%) of older adults aged 60 years and over globally are not doing sufficient physical activity [13].

In addition to age-based disparities in physical activity, disparities exist according to socioeconomic status (SES). SES is used to describe multiple factors relating to the absolute and/or relative social position of individuals. It can be measured in a number of different ways, at the individual level, including income, levels of education, occupation, and area level deprivation of the home address [14, 15]. Whilst individual measures such as educational level and objective income are most commonly used to measure the socioeconomic position in research with older adult populations [16], area level measures are also useful where health inequalities can occur because of factors related to area level deprivation and the social environment [16]. This is important in physical activity research as factors such as access to green space and safety of the neighbourhood environment are important determinants of engagement in physical activity [17, 18]. Area level measures are also easy to obtain and widely available in large national datasets [16]. Both individual-level and area-level SES are associated with levels of engagement in leisure-time physical activity amongst older adults [19].

Numerous studies across Europe, North America and Australia show lower levels of physical activity, particularly during leisure time, amongst individuals of lower SES [20–22] and these disparities exacerbate with increasing age [22]. Older adults of lower SES are therefore at particular risk, sitting at the intersection of two major determinants of lower engagement in physical activity (increased age and socioeconomic disadvantage), and represent a key target group for public health interventions with the aim of increasing physical activity.

During the COVID-19 pandemic, opportunities to engage in physical activity were reduced due to public health restrictions to help prevent the spread of the virus. Whilst these measures affected the entire population, older adults were disproportionately affected as their vulnerability to COVID-19 meant they were asked to stay at home/shield for longer, reducing their opportunities for social interaction and leading to fear at resuming their normal activities upon the lifting of measures [23], and putting them at increased risk of problems associated with prolonged physical inactivity. There was also higher compliance with COVID measures and restrictions in the UK amongst older adults (aged 60 years and over) compared to younger adults [24, 25]. Older adults may have also been less likely to participate in remote opportunities to engage in physical activity, such as online fitness classes, because of digital exclusion. Internet usage declines with age; data from January to March 2020, found that 86% of 65–74-year-olds in the UK were recent internet users, and this dropped to 54% in those aged 75 years and over, compared to 99% in adults aged 16–44 years [26]. Whilst the latest report from the UK’s Office of Communications (Ofcom) using data from 2024, shows that of those without access to the internet at home, 56% were aged 65 years and above [27]. The same report also showed that those aged 85 years or over were 8 times more likely to be non-internet users (i.e. no usage at home or outside of the home) and those aged 75–84 years old twice as likely to be non-internet users. Research from the USA and Europe also shows socioeconomic disparities in internet usage amongst older adults, with those of lower SES having lower and less broad usage [28, 29], and persistence of these disparities during the pandemic [30].

Research examining physical activity levels amongst older adults demonstrated a persistence of socioeconomic disparities during the pandemic, with studies from the UK and Japan showing older adults of lower SES performing less physical activity compared to those of higher SES [31–33]. Other research from the UK and South Korea even identified a widening of these disparities in activity levels [34, 35]. Whilst evidence demonstrates a recovery of physical activity to pre-pandemic levels, amongst the general population of older adults, this is not the case for lower SES population groups. For example, data from the national survey of physical activity levels in England, demonstrates that the percentage of older adults achieving at least 150 min of moderate intensity physical activity per week, increased post-pandemic to pre-pandemic levels and continues to grow [36]. However, the survey highlights increasing inequalities in physical activity levels between those from more affluent and less affluent groups, as categorised by social grade based on occupation, and between those living in the least and most deprived areas [36]. It is therefore important to develop appropriate interventions to help with post-pandemic recovery of physical activity levels, and to increase physical activity amongst older adults from lower SES groups more broadly, to prevent further widening of any socioeconomic inequalities.

A recent systematic review, whose included studies were primarily from North America and Europe, found mixed effectiveness of existing interventions at increasing levels of physical activity amongst this group; positive effects were found for increases in physical activity for a purpose (i.e. walking for transport) but not for leisure, mixed effects on objectively measured physical activity, and no effects on self-reported total physical activity or muscle strengthening and flexibility activities [37]. The same review also examined the acceptability of physical activity interventions amongst older adults of lower SES. Acceptability is defined as the extent to which an intervention is considered appropriate by those delivering or receiving it, and is a key concept to consider at all stages of intervention development, as it has important impact for overall intervention uptake and effectiveness [38]. The review found that engaging in physical activity was perceived as having many benefits, not just those related to physical health but also psychological and social benefits [37]. Social familiarity was important, being amongst peers with whom they shared characteristics with, both in relation to other participants but also those delivering the intervention, facilitated engagement with physical activity [37]. The compatibility of physical activity interventions with the lifestyles of older adults with lower SES was also important to acceptability. Using convenient and familiar locations were facilitators, whilst participants often described competing commitments and demands on their time as barriers to engaging with physical activity interventions. Combining physical activity with other activities they were already doing helped to overcome these barriers [37]. Data collected from most of the studies included in the review were conducted prior to the COVID-19 pandemic, with only two studies completing data collection into the first few months of 2020, whilst COVID-19 lockdown restrictions were still in place.

Other primary qualitative research looking at the acceptability of engaging in physical activity, in the community pre-pandemic, amongst older adults experiencing socioeconomic disadvantage in Australia, found that barriers included deteriorating health, a lack of belonging and loss of motivation [39]. Whilst a lack of availability in community services and resources, as well as a negative social environment (related to a lack of feeling of community belonging, and feeling vulnerable to violence and crime), were barriers to reducing sedentary behaviour in England [40]. Another study in England found that older adults living in lower SES areas (those defined as having higher levels of older adults experiencing deprivation) also reported feeling undervalued and disadvantaged when comparing themselves to older adults living in higher SES areas, due to factors such as lack of services and loss of local facilities [41]. Whilst physical activity was perceived as more acceptable when activities are enjoyable, familiar and address multiple needs, such as social connection and leisure interests [41].

However, data from previous research looking at the acceptability of physical activity amongst older adults from lower SES groups, was mainly collected prior to the COVID-19 pandemic, and therefore does not offer insights since the pandemic. To inform the development of effective interventions to support recovery of physical activity following the pandemic, and to promote increased physical activity amongst this population more broadly, we first need a greater understanding of the impact of the pandemic on engagement in physical activity amongst this group. To date and to the best of the authors’ knowledge no research has explored the acceptability of engaging in physical activity after the COVID-19 pandemic, amongst older adults from lower SES groups. The present qualitative study, therefore, aims to explore the views of older adults residing in areas of high socioeconomic deprivation around the acceptability of engaging in physical activity, in the aftermath of the COVID-19 pandemic.

## Methods

### Participants and recruitment

Community-dwelling older adults, aged 65 years and over, and living independently, in areas with high levels of socioeconomic deprivation, in Manchester, England were eligible. Individual measures of SES (such as income, wealth, or education) were not used as eligibility criteria as we used place-based definitions of deprivation as a proxy for SES. Participants were recruited using purposive and snowball sampling. Manchester is a local authority with one of the youngest average ages in England (median age of 31 years compared to the national median age of 40 years). Despite a large population of students and young professionals living in the area and an increase in recent developments, a sizeable proportion of the older adults living in Manchester experience socioeconomic deprivation. Manchester is the 4th highest local authority district nationally for older adults experiencing socioeconomic deprivation; 34% of people aged 60 and over live in income deprived households (compared to the national average of 18%) [42].

The Income Deprivation Affecting Older People Index (IDAOPI) was taken from the Index of Multiple Deprivation (IMD) 2019 dataset [42] and was used to select areas with the highest percentages of income deprived older adults in the Manchester Local Authority to recruit participants from. The IDAOPI is a subset of the Income Deprivation domain of the IMD and measures the proportion of all those aged 60 and over who experience income deprivation. Areas selected for recruitment were amongst the 10% most deprived in England. Participants were recruited from these areas via local community groups, age-related charities and ageing networks. The lead author (DH) contacted community group leaders via email and telephone and visited local community centres and groups in-person. Advertising posters were also placed in local community spaces. Snowball sampling was also used, where participants who had already taken part helped to identify additional potential participants.

### Data collection

Semi-structured one-to-one interviews lasting up to one hour, were conducted face-to-face (in a private room on the university campus or in a public place near to the participant’s home address, such as a community centre) or online via Zoom according to participant preference. Prior to interview, the eligibility of those who responded to the study adverts was assessed and participant information sheets given to participants. Informed consent was collected prior to data collection.

Following consent, participants were asked to complete two structured questionnaires immediately before the interview. A short demographic questionnaire was used to capture data relating to participants’ age, gender, ethnicity, educational level, occupation (either current or prior to retirement) and health status (assessed using the one-item Self-Rated Health [43]. The Physical Activity Scale for the Elderly (PASE) questionnaire [44] was used to measure participants’ physical activity levels. This brief measure is reliable and valid for measuring physical activity in adults aged 65 years and over and covers physical activity performed for leisure, around the house and at work [44]. For the purposes of this study to determine whether participants were meeting physical activity guidelines (≥ 150 min of moderate intensity or ≥ 75 min of vigorous intensity physical activity per week) during leisure time, we looked at the amount of time per week participants reported spending in moderate or vigorous recreational activities.

The semi-structured interviews were guided by a topic guide (Additional file 1), which was developed from a previous qualitative study exploring the acceptability of physical activity amongst older adults living in socioeconomically deprived areas [41] and adapted to add in questions regarding the COVID-19 pandemic. Questions were also refined in consultation with public contributors prior to gaining study ethical approval. For example, the public contributors thought it was important to acknowledge how family members may encourage physical activity, and as a result the following question was added to the topic guide “What influence do others have on how physically active you are?” Interview questions focused on participants’ levels of activity, how they felt about physical activity, availability of activities in their local area, barriers and facilitators to engaging in physical activity, and whether any of these factors had changed compared to before the COVID-19 pandemic. A small pilot (*n* = 2) was carried out at the start of data collection to check that the interview schedule was appropriate, that questions made sense to participants and to check interview timings. These pilot interviews were included as part of the total sample. Participants received a shopping voucher as an acknowledgement for their participation. Interviews were conducted and audio-recorded by the primary researcher (DH) and transcribed *ad verbatim* by a professional company. Data were collected between March and December 2024. The principles of information power [45] were used to determine the ceasing of data collection. Information power was achieved through a combination of having a narrow study aim, purposive sampling strategy targeting recruitment in specific areas, as well as aiming for a diverse sample in terms of age range, gender and ethnicity, and conducting in-depth interviews guided by a focused topic guide with open-ended questions. While the sample size was relatively small (*n* = 16), the strong quality of the dialogue in the interviews and the in-depth data analysis allowed for a comprehensive understanding of the research question.

### Data analysis

Interview transcripts were exported into NVivo Pro14 software for data management. Data was analysed using reflexive thematic analysis, which is a method for developing, analysing and interpreting patterns across qualitative datasets [46]. Reflexive thematic analysis emphasises the role of researcher subjectivity as the primary tool for analysis [47]. The analytic outputs of this method are themes, defined as specific patterns of shared meaning across the dataset, each organised around a central concept [48]. Themes are actively generated by the researcher through systematic and deep engagement with the dataset [47].

Data analysis followed six phases: (1) familiarisation with the data, (2) coding, (3) generating initial themes, (4) developing and reviewing themes, (5) refining, defining and naming themes and (6) writing up the report. An inductive approach was used, whereby coding and themes were driven by the data to best represent meaning as communicated by participants to capture their experiences, rather than being developed through the lens of pre-existing theories which can provide a less rich description of the data [49]. Firstly, the lead author (DH) who conducted the interviews became familiarised with the data through listening to the interview recordings, reading and re-reading the transcripts. The same researcher also then coded the data. Coding was performed initially at the semantic level to explore surface level meaning. These initial codes were then discussed with two other authors (AG and AH) who provided further insights to sense-check ideas and consider different, richer interpretations of meaning within the data. These discussions informed the next stage of coding at a more latent level to explore more implicit meanings from participants’ words. The lead author then generated initial themes by organising codes together into meaningful patterns, which were discussed and reviewed with the research team. All authors critically reviewed the themes, including the refining, defining and naming of these. The consolidated criteria for reporting qualitative studies (COREQ)−32 items [50] was followed for reporting this study, the completed checklist is available in Additional file 2.

### Researcher characteristics and reflexivity

The lead author (DH) is a White British female PhD candidate from the North West of England with a background in psychology. Her research interests include physical activity, behaviour change and health inequalities. She has previous experience of qualitative research methods. The research team further consisted of the lead author’s PhD supervisors who are multi-disciplinary, with backgrounds in clinical exercise physiology in ageing populations (AG), healthy ageing and social gerontology (AH), psychology (EE) and health economics and inequalities (LM). The research team had no prior relationship with participants prior to the study.

## Results

Sixteen interviews were conducted. Participants’ ages ranged from 65 to 98 years, with an average age of 75 years. Eight (50%) were male and eight (50%) were female. Twelve (75%) were not meeting current physical activity guidelines (≥ 150 min of moderate intensity or ≥ 75 min of vigorous intensity physical activity per week) during leisure time, these were categorised as ‘insufficiently active’, whilst four (25%) met the guidelines and were categorised as ‘sufficiently active’. Nine (57%) were educated to degree level. Eight (50%) had medium level skill occupations based on International Standard Classification of Occupations-08 (ISCO-08) currently or prior to retirement, these occupations included Plant & Machine Operators & Assemblers, Service & Sales Workers, and Clerical Support Workers. Four (25%) had high skill level occupations (Professionals and Technicians & Associate Professionals). Further details of all participant characteristics are found in Table 1. Six themes were generated from the data analysis: (1) The COVID-19 pandemic - a teachable moment for physical activity, (2) Physical activity - for purpose or pleasure?, (3) Focus on what you can do rather than what you can’t do,, (4) Opportunities to be physically active (which consisted of two sub-themes: Place and Time), (5) Importance of social environment, and (6) Physical activity messaging and perceptions. An overview of themes and sub-themes, alongside corresponding codes is found in Table 2.

**Table 1:** Participant characteristics

Characteristic	Total (*N* = 16)
Age in years	
Mean (SD)	75.2 (9.4)
Gender	
Male	8 (50%)
Female	8 (50%)
Ethnicity	
White British	9 (56%)
White Other	1 (6%)
Chinese	3 (19%)
Pakistani	2 (13%)
Japanese	1 (6%)
Highest level of education	
Did not complete secondary school	3 (19%)
Completed secondary school	4 (25%)
Post-secondary school	0 (0%)
Bachelor’s degree	3 (19%)
Postgraduate degree	6 (38%)
Occupation	
Professionals	3 (19%)
Technicians & Associate Professionals	1 (6%)
Clerical Support Workers	1 (6%)
Service & Sales Workers	3 (19%)
Plant & Machine Operators & Assemblers	4 (25%)
Housewife	2 (13%)
Not Reported	2 (13%)
Self-rated health status	
Excellent	0 (0%)
Very Good	4 (25%)
Good	7 (44%)
Fair	4 (25%)
Poor	1 (6%)
Meeting physical activity guidelines	
Yes (sufficiently active)	4 (25%)
No (insufficiently active)	12 (75%)

**Table 2:** Overview of themes, sub-themes and codes

Themes	Sub-themes	Codes
The COVID-19 pandemic - a teachable moment for physical activity		• Decrease in physical activity during lockdowns • Making up for lost time • Widening of physical activity opportunities • Increased awareness of importance of physical activity for health
Physical activity - for purpose or pleasure?		• Purposeful physical activity • Enjoyment is important • Formal exercise is not fun • Combining physical activity with leisure interests
Focus on what you can do rather than what you can’t do		• Change in physical activity with age • Physical health limitations • Adapting physical activity to own capabilities
Opportunities to be physically active	• Place • Time	*Place* • Access to green space • Preference for outdoor activities • Poor weather is a barrier • Neighbourhood environment is a barrier • Access to facilities & activities • Need for age friendly spaces • Dislike of gyms *Time* • Competing commitments • Regular physical activity routine is important
Importance of social environment		• Social connectivity is a motivator • Preference for group activities • Encouragement and support from family & friends
Physical activity messaging and perceptions		• Physical activity is important • Lack of awareness of physical activity guidelines • Messaging not relevant to me • Physical activity need not be complicated

### The COVID-19 pandemic - a teachable moment for physical activity

Participants reflected on how the COVID-19 pandemic led to changes in not only their levels of physical activity, but also their attitudes towards physical activity. Whilst participants described initial negative effects of the pandemic on their levels of physical activity, there was sense of a more promising outlook moving away from the pandemic, with an increased motivation to do more physical activity, and recognition of the importance of physical activity for health and wellbeing.

Many participants described how the national lockdown policies had restricted their levels of physical activity. Some participants also described how even after lockdown restrictions were lifted, they had almost become conditioned into staying in the house, and this was mentally challenging to overcome and get back into their normal activities.

“There’s a psychological aspect of getting used to being inside and at home…it took a long time to get into doing the physical…the actual getting out and going walking or going to groups. It took quite a while to do that.” (Male, 67 years, good health, insufficiently active).

Despite initial negative effects on their levels of physical activity during the pandemic, more positive effects were also reported. Some participants described how their levels of physical activity had increased post-pandemic; this was often linked with a desire to make up for lost time during lockdown restrictions, when opportunities to be physically active were restricted.

“I would say I probably do more now, though, than I did before COVID. Because you couldn’t do enough when COVID was on, hardly at all. A few exercises in your own house. That’s all you could do.” (Male, 82 years, good health, insufficiently active).

Some participants also reported how the pandemic led them to apply different ways of performing physical activity. For example, one participant used a deliberate strategy when food shopping to minimise the amount of time spent outside the house, which they compensated for by performing more physical activity at home.

“During the pandemic, I stayed active by walking around the house. Every time I went out, I’d buy a bit more food to avoid going out too often. But I still made sure to do plenty of exercise at home.” (Female, 98 years, good health, sufficiently active).

Another example was using digital tools to engage with physical activity opportunities from across the world, something which was a novel experience as reflected on by one participant and not something they had even considered prior to the pandemic.

“So it’s all the classes that I’m really grateful that teachers who made efforts to find Zoom… So that’s something that COVID brought in for good effect…And then also online, it’s more like global. I didn’t realise, that’s pretty amazing how much - I never thought of having a Qigong class from the West Coast of America.” (Female, 68 years, good health, sufficiently active).

Many participants reported how the pandemic had led to an increased awareness of the importance of physical activity for their health, and in turn an increased motivation to be physically active post-pandemic. This was often cited amongst those who were insufficiently active. This sentiment was expressed via a perceived broadening of knowledge that increasing physical activity is important amongst older adults more generally in the wake of the pandemic.

“Before the pandemic, people didn’t exercise much. But now, everyone understands that as we get older, we need to exercise more to stay healthy. So, I think it has made a difference. It’s definitely important to exercise more.” (Female, 73 years, good health, insufficiently active).

This was also coupled with strong individual statements of determination to do more physical activity.

“It’s [the COVID-19 pandemic] made me realise how important it [physical activity] is, so I won’t let anything stop me doing it in the future.” (Male, 73 years, fair health, insufficiently active).

The pandemic had less of an effect on the desire to increase physical activity amongst those who were sufficiently active, but these participants still experienced a shift in outlook on the importance of leading a healthy lifestyle in general, due to the threat to life that COVID-19 posed.

“You realise it’s all very precious, and people lost people didn’t they? So, I think it changed my view of life itself, and that, you know, you want a healthy life…but it’s not that I’ve done nothing, that I was a slob all the way, and then suddenly the pandemic came, and I went right… So to a certain extent, kind of more philosophically in outlook rather than suddenly, right, I’m going to double the amount [of physical activity] that I’m doing” (Female, 67 years, very good health, sufficiently active).

### Physical activity - for purpose or pleasure?

Many participants described how a lot of their engagement in physical activity was often linked with purposeful tasks. One example was transport:

“I cycle every day of my life – it’s my mode of transport, it’s nothing to do with exercise…It just gets you from A to B…my physical activity is just the way I get around.” (Male, 67 years, very good health, sufficiently active).

This quote shows how physical activity was sometimes a byproduct rather than an intentional activity on its own. For other participants physical activity achieved by purposeful means was more intentional, and seen as an important component of their overall physical activity regime. One participant had even increased the amount of housework they were performing when lockdown restrictions meant opportunities to move outside of the home were reduced.

“The time spent exercising outdoors has decreased. At home, I vacuum, mop, and do cleaning—that’s exercise too. I used to vacuum once a week, but now I do it every day.” (Female, 98 years, good health, sufficiently active).

Whilst for others household activities were their sole source of physical activity, describing a preference for purposeful physical activity rather than exercise for the sake of it.

“I’ve never been one for exercising. The only exercise I like to do is walking and keeping myself working around the house…And your shopping, you know, the main things.” (Female, 78 years, fair health, insufficiently active).

Sometimes rather than household tasks being conceptualised as a form of physical activity themselves, participants used this time as an opportunity to perform other physical activity.

“Some exercises I do while cooking. When I’m waiting for the food to cook, I take the opportunity to move around a bit.” (Female, 73 years, good health, insufficiently active).

Caring for others was another contributor towards participants’ engagement in physical activity.

“I’m running around with grand-daughters and mothers, in and out of the house, up and down the stairs.” (Female, 67 years, very good health, sufficiently active).

Despite participants often describing how a lot of their physical activity was achieved through non-leisure activities, many participants also reflected that a sense of enjoyment and finding something to do for pleasure was important to them when engaging in physical activity.

“No good taking up gardening, if you don’t like gardening. No good going bowling… Try it for a couple of weeks, if it’s not for you, don’t do it… you have to find something you like doing” (Male, 82 years, good health, insufficiently active).

This sentiment was also echoed by a resistance to engage in physical activity when it felt like an obligation.

“So, I sort of, turned down that recommendation to join a gym. Partly, I suppose, when people are forced to do things, they don’t do them. I can imagine that someone who is told to go to a gym probably wouldn’t, just because they’re told to do it. So, I think it’s better to ease people into activity and make it a pleasure…it’s not a duty in that sense. It’s something you do, and if you do something you enjoy, the benefits are better I would think.” (Male, 75 years, good health, insufficiently active).

Formal exercise classes or going to the gym were often seen as lacking a sense of fun or engagement amongst participants, and hence many participants were not interested in such activities, which were often seen as not pleasurable.

“Whereas, I’ve gone to classes in gyms, where, yeah, you’ve got a sweat on, but was it enjoyable? Oh, there was one I had to stop going to, ‘cause the woman, she had the music on really loud, and it wasn’t my type of pop, it was just a racket, horrible, and she’d be shouting…shrieking the instructions and you ended up with a headache…And it’s just not really good, you didn’t get any pleasure from it, even though you’ve worked out” (Female, 67 years, very good health, sufficiently active).

Participants’ other leisure interests were also seen as a facilitator to helping them engage in more physical activity for some. Examples included either combining walking with a specific activity, “where there’s a bit of interest, a bit of bird watching” (Male, 74 years, fair health, insufficiently active), or to explore “somewhere that they haven’t been before” (Male, 75 years, good health, insufficiently active).

Another participant also described how they liked to combine their exercise sessions at the leisure centre alongside other hobbies.

“But I tend to go and study something in the café…you can see some people just go in and go hell for leather, running up and down for half an hour…But I do like to go and study something in the café.” (Female, 67 years, very good health, sufficiently active).

### Focus on what you can do rather than what you can’t do

Many participants described how their physical activity had changed with age. Sometimes this was expressed as a form of internalised ageism, whereby participants stated that they felt too old to be engaging in certain types of physical activity that they used to enjoy.

“You know, getting a bit old to do aerobics and stuff, bending your bones. No, I’m not into anything like that. I did do that when I was 17, not now, when I’m 70-odd.” (Male, 74 years, fair health, insufficiently active).

Or the myth that they should not engage in too much physical activity in general, simply because of their biological age: “I can’t be too active, I am old already haha.” (Female, 98 years, good health, sufficiently active). However, participants who were insufficiently active, frequently expressed that physical health limitations that accompanied older age were key barriers to engaging in physical activity. For some participants walking was difficult.

“...we used to walk miles but then it just goes doesn’t it. The aches and pains you get, you don’t feel like doing it.” (Male, 74 years, fair health, insufficiently active).

Whilst for others it was lifting weights that proved problematic.

“…heavy exercise is difficult for me… Yeah, lifting… Yeah, difficult… Because this side is very weak.” (Female, 66 years, poor health, insufficiently active).

As a result of these physical health limitations, many participants described adapting their level of physical activity in accordance with their own physical capabilities. This was expressed in different ways. One example was changing the type of physical activity performed.

“There’s nothing I can do about it because I can’t walk properly. But I do exercises at home, like walking on my tiptoes in place, and I also massage my shoulders, hips, arms, and so on against the wall. I keep exercising at home without stopping.” (Female, 73 years, good health, insufficiently active).

Another example was reducing the intensity of physical activity.

“And when I first came, I thought I’ll have a go in the gymnasium on the treadmill… I found it a bit arduous. So, no, too much for me, yeah. And they’ve got a cycling machine in there, I thought I’ll try that, good for my legs, but I’m afraid it was hard work… So, I gave it up. So, I do the more gentle side of the exercises” (Male, 90 years, very good health, insufficiently active).

Whilst for others it was having rest days away from being physically active.

“…but like I say, if you feel you can’t do it, you shouldn’t do it. Do it the next day if you feel better. You shouldn’t do above what you can do. You will only end up being worse off.” (Male, 82 years, good health, insufficiently active).

### Opportunities to be physically active

#### Sub-theme: place

Many participants talked about the importance of having access to green space. This was reflected on more generally, and particularly during the pandemic when lockdown restrictions were in place. Some participants saw value in having access to green space for specific activities.

“I live between two lovely parks, so, they offer a range of activities, physical activities, around gardening, tree maintenance, bird watching all sorts of things and there’s, like, a gardening project involved in both parks which I have some dealings with” (Male, 67 years, very good health, sufficiently active).

Whilst for others it was simply about having the opportunity to walk around.

“I’m very lucky, I think, because I’ve got two parks on either side, so during lockdown, when we had an hour a day, I loved it, I’d work all morning at home, and then lunchtime, we would go round both parks, and it filled the hour, at a good brisk pace.” (Female, 67 years, very good health, sufficiently active).

The importance of maintaining access to green space was discussed, amidst threats to its loss from proposed developments.

“But now I thought COVID made people understand how important to keep the local green space…because you can’t go further out to the countryside by car, there are some lockdowns…So that’s why I’m just now involved with a community, a big community project to keep our green space from the housing development.” (Female, 68 years, good health, sufficiently active).

Many participants described a preference for performing physical activity outdoors and spending time in nature.

“Oh, and camping, I love camping and glamping, and you get quite fit doing that, because you don’t just go off on walks but you’re on your feet a lot…you feel out in nature a lot, and I love it. I love waking up in a field, and listening to birds and stuff.” (Female, 67 years, very good health, sufficiently active).

However, some participants cited barriers to performing physical activity outdoors, one of which was poor weather, which was also linked to a fear of falling by one participant. Negative issues related to the neighbourhood environment were also described as barriers to engaging in physical activity outside, mainly related to safety fears.

“…we have a bigger area…but it’s a bit far away from my place and I don’t feel necessarily safe.” (Female, 68 years, good health, sufficiently active).

“…if your neighbourhood is not very attractive for whatever reason…it could be litter and also there might be…if there’s a fear of or a reality of street crime, people won’t perhaps want to venture out the house and go for walks and stuff…” (Male, 67 years, very good health, sufficiently active).

With regards to other opportunities to engage in physical activity, many participants reported that they felt they had good access to facilities and activities, such as leisure centres, organised physical activity classes and groups.

“Manchester’s blessed with leisure…we can access leisure centres to do all those things” (Male, 67 years, very good health, sufficiently active).

Some participants were taking advantage of these opportunities and were actively using them.

“Well, here we have an exercise class every Monday, it’s stretch and flex exercises. And I go to those every Monday afternoon, for one hour… It’s very good. And then on Fridays here we have Tai Chi.” (Male, 90 years, very good health, insufficiently active).

However, for others they were not presently using facilities despite being aware of them.

“I mean, the swimming baths is over there, but…I haven’t been recently” (Female, 84 years, good health, insufficiently active).

Some participants also reflected on the importance of ensuring that spaces and activities are age friendly to encourage more older adults to engage with them. One participant who was using a leisure centre thought that this needed to attract more older adults generally.

“I always thought they needed to appeal more to the older people, you know young people… but I don’t think enough older people do exercise, still.” (Female, 67 years, very good health, sufficiently active).

Another participant despite enjoying swimming, expressed that facilities could be better catered to older adults.

“See, you go swimming, I like swimming but the swimming pool’s that cold for an older person, you have a bloody heart attack just getting in them” (Male, 74 years, fair health, insufficiently active).

Whilst another participant expressed that exercise classes which he had attended felt more age appropriate by using music that was relatable.

“She used to play music for the activities…1950s, 1960 s, sort of rock and roll stuff and that, 1960 s pop music and everything.” (Male, 82 years, good health, insufficiently active).

One place which was not popular amongst participants was gyms, with many expressing a dislike and/or disinterest for using these spaces. There were several reasons given for this, including that they were indoors, there was a sense of social pressure to keep up with other people and an overly serious atmosphere.

“And she recommended going to a gym, but I don’t fancy going to a gym…because they’re indoors…And they’re quite intensive and you feel you’ve got to keep up with the people next to you, and they take things very seriously.” (Male, 75 years, good health, insufficiently active).

Another reason was that gyms offered little opportunity for social connection.

“I think sometimes doing it [physical activity] with other people is also important…Some people just do it at gym. That’s why I’m not interested in gym.” (Female, 68 years, good health, sufficiently active).

#### Sub-theme: time

Many participants described how limited time was often a barrier to engaging in physical activity, due to various competing commitments such as caring for others, work, household chores and attending medical appointments. Time was cited as a barrier to both those who were already meeting physical activity guidelines and those who were not. Some participants expressed a need for keeping to a regular routine for engaging in physical activity, ensuring that time is set aside specifically for physical activity.

For those who were insufficiently active, physical activity was seen as secondary to other commitments.

“…when you have to go to the hospital and doctors and stuff like that. You think, I’ll go over the garden and then you don’t go over the garden because you have something else, and stuff like that, you know. So sometimes it’s not easy to fit it in.” (Male, 82 years, good health, insufficiently active).

These participants also felt like they were less able to move their other commitments and so needed physical activity to fit around them.

“If somebody set up a class, like I say, like we had before, I would do that, yes. I think it was on a Tuesday, Tuesday morning about 9:00 o’clock, 9:30 to about 10:30. If someone would do that, because being that time of day don’t interfere with anything else…you wouldn’t be going out before that time really. So, it didn’t really stop you from doing other things” (Male, 82 years, good health, insufficiently active).

Whereas those who were sufficiently active felt they had more agency to balance physical activity and their other commitments.

“I think it’s the balance is because you’ve got to do some certain housework as well…So I think it’s at the moment balancing and then including in the shopping and all these things.” (Female, 68 years, good health, sufficiently active).

These participants also tended to prioritise time for physical activity.

“For exercise, I swim twice a week…I mean, that’s more obviously leisure in that I’ve got to get myself to take time out of the day to get to the leisure centre to do swimming.” (Male, 67 years, very good health, sufficiently active).

There was also a desire amongst those who were already meeting physical activity guidelines to increase their levels of physical activity further if their other commitments were to reduce.

“Elderly parent, own kids and although they’re grown up they still need some attention, and then grandchild and things, and then the wider family. So I suppose it’s all that. Would I do more…? Yes, I would do more, if I were freed up from that.” (Female, 67 years, very good health, sufficiently active).

### Importance of social environment

Many participants described a preference for group physical activities over exercising alone, with social connectivity often seen as a motivator for engaging in physical activity.

“The running, I don’t run on my own – some people do – but the Park Run is a very sociable thing…it gets you up on Saturday morning and you spend half an hour with people and then you go for a coffee afterwards. So, that’s quite a social thing. I wouldn’t dream of…no, I have in the past occasionally run round the park on my own, but for that, that activity, in fact it’s a brilliant concept is the Park Run, it is a very social thing and you get exercise.” (Male, 67 years, very good health, sufficiently active).

Whilst for others who were not currently engaging in group physical activity, it was seen as an attractive prospect to consider.

“I have to motivate myself ’cause, like, I live on my own with the cat. Like, it might be a good thing to…if I can fit it in my timetable, to go out with the group…to go to where there are groups of people, like the walking group, or maybe even the cycling group. Yeah, I think that would help.” (Male, 67 years, good health, insufficiently active).

Group physical activity in general was also seen as more fun than individual physical activity:

“So, doing it on your own it’s a bit…it can be a bit boring, collectively it’s better.” (Male, 90 years, very good health, insufficiently active).

The social benefits obtained from physical activity were also sometimes seen as more of a priority to participants than physical health benefits.

“But to me, walking is a leisure activity for fun. And when you’re out with a group, you don’t say, you know, we’re doing this walk to get healthy. We’re doing this walk to speak to each other.” (Male, 75 years, good health, insufficiently active).

Many participants also reported how encouragement and support from family and friends helped to facilitate their engagement in physical activity. This was expressed through general words of encouragement from others.

“So yeah, and I do try and get out of my wheelchair…because I can walk a little bit, as you’ve seen earlier. I just try and do a little bit, and everyone in the cafés knows who I am and, sort of, say, come on, get up and move around, you know.” (Male, 73 years, fair health, insufficiently active).

In addition to others providing more direct instructions.

“But my wife motivates me, she’s a retired nurse, so she tells me exactly what to do.” (Male, 90 years, very good health, insufficiently active).

Sometimes this support also helped with initiating engagement in new activities.

“I just think people asking you if you want to try this or if you want to try that. It’s like the gardening over the allotment…the next door neighbour, he said, why don’t you come over to the allotment?” (Male, 82 years, good health, insufficiently active).

One participant, who was the oldest in the sample, even expressed how they themselves provided encouragement for others around them to engage in physical activity.

“I often invite my neighbours to join me for exercise…I take them to physical therapy, and recently I’ve brought another one along.” (Female, 98 years, good health, sufficiently active).

### Physical activity messaging and perceptions

Most participants expressed awareness of the importance of keeping physically active. Its general importance for the whole population was reflected upon.

“...I mean physical activity is just…we have to do it every day, unless you’re paralysed, quadriplegic, it’s something you have to do just every day just to function as a human, right?” (Male, 67 years, very good health, sufficiently active).

Many participants also reflected on the increasing importance of physical activity in older age. This was described for different reasons; one was its importance for physical health.

“…exercise is really good for the body. For elderly people, it’s especially important for their health, as it helps them feel healthy and safe.” (Female, 73 years, good health, insufficiently active).

Another reason was to prevent more general functional decline in older age.

“I’ve realised it’s actually vital when people get into 70 s and 80 s it seems more important that they keep moving. Because once you sit down for a day…once you slow down, it’s not possible to get back really. So, anyone who is aging, certainly when people retire, there’s the temptation just to, sort of, ease up. But actually, when you retire you should do more” (Male, 75 years, good health, insufficiently active).

In addition, helping to maintain cognitive function was also seen as an important reason to be physically active.

“Well, you imagine if you sit down all day for five days a week, four days a week, so it’s a thing that you keep your brain active as well. You have to keep your brain active as well as your body, you know. Mainly I think a lot of it is when people retire. They don’t keep their brain active.” (Male, 75 years, good health, insufficiently active).

However, there was a lack of awareness and some confusion regarding the level of physical activity that is recommended for older adults. Whilst some participants felt that there must be some guidelines, they were not very aware of the content of these. One participant even discussed how guidance was not as communicated widely to the public compared with her country of birth.

“I didn’t know about the guidelines. I’m sure there is some, but I didn’t know that. I don’t think we have, so it’s a government guidelines…in Japan, it’s very much we have a health news is coming through the door because my family, my son’s living in Japan so when I visit, I always get and they’re talking about all this exercises and then classes available very much in the community…So when you say guidelines, I’m sure there’s a have half an hour walking or outside exercise, I’m sure there’s some recommendations, but unfortunately, I don’t know about that.” (Female, 68 years, good health, sufficiently active).

Whereas other participants were not aware of the existence of physical activity guidelines, however they did recognise the importance of regular physical activity.

“No one guides us. But I only know that it’s good to do exercise every day.” (Male, 76 years, very good health, insufficiently active).

One participant was more familiar with regards to the amount of time one should spend being physically active, however they perceived public health messaging regarding physical activity to be more focused on children rather than older adults.

“Oh god, older adults. Gosh, what is it? Is it 30 minutes activity five times a week? I can’t remember what the…perhaps the figures change. And there’s more of a stress on, is it children and particularly related to childhood obesity.” (Male, 67 years, very good health, sufficiently active).

One participant also described how imagery and messaging used in guidance are often not relatable.

“I think it’s very hard because when they show you these pictures of people, shown doing the exercise, they look about seven, weigh about seven stone and like a Russian athlete. Not someone that’s over 70 and overweight. The instructions aren’t made for a fat guy, they’re made for a thin person, already.” (Male, 74 years, fair health, insufficiently active).

Some participants also reflected on how physical activity need not be complex (e.g. requiring lots of equipment) or cost money. These participants tended to be those who were already meeting physical activity guidelines. They reflected on how physical activity can be simplistic and easily fit into their lives, and that more traditional definitions or perceptions of physical activity may have been overcomplicated.

“…physical activities, my definition is more bigger than what people tend to think. It’s not just you have to have a gym to do that. You can just create yourself that simple movement or simple connection with people, you can create physical activity or physical benefit.” (Female, 68 years, good health, sufficiently active).

“The concept of you drive to a gym…and then you exercise on a treadmill, it just seems bonkers…everything is commercialised. They commercialise the activity, well, why do you need to? Just walk out your door, you walk to the park, you get on a bike, there’s no need for that.” (Male, 67 years, very good health, sufficiently active).

## Discussion

This study aimed to investigate the views of older adults residing in areas of high socioeconomic deprivation, around the acceptability of engaging in physical activity in the aftermath of the COVID-19 pandemic. Firstly, we found that the COVID-19 pandemic was seen by many of the older adults as a teachable moment with regards to physical activity. There was an increased enthusiasm to do more physical activity post-pandemic, with a desire to make up for the time lost when physical activity was restricted, exploring new opportunities to be physically active around the home, and increased awareness of the sense of the importance of physical activity for health.

Teachable moments are defined as health events or situations which lead individuals to engage in positive health behaviour change, they can be both naturally occurring events or can be created through clinician–patient interactions [51, 52]. According to a proposed conceptual framework [51], events or situations which instigate health behaviour change and are determined teachable moments are defined by three characteristics: (1) an increase in risk perception, (2) an affective response that is strong enough to promote behaviour change and (3) a change in a person’s self-concept in order to stimulate the adoption of healthier behaviours. These concepts were evidenced in the current study. Participants described an awareness of a heightened risk to their health of catching COVID-19, and a strong affective response of fear at leaving their house during the height of the pandemic. In addition, a change in self-concept was demonstrated, through a shift in emphasis on the sense of importance of being more physically active and leading a healthy lifestyle.

Other research has also demonstrated that the COVID-19 pandemic served as a teachable moment, and led to intentions to increase physical activity [53, 54], with 34% of participants amongst a general adult sample of over 900 respondents from Brazil [54], and 25% of participants amongst over 800 cardiovascular disease (CVD) patients from the Netherlands [53], reporting increased intentions to increase physical activity as a consequence of the pandemic. Predictors of increased intentions to improve physical activity were a higher affective response [54], evaluated by measuring participants’ worries, stress and negative feelings during COVID-19 restrictions, and a change in self-concept [53], measured via two items focused on the level to which the COVID-19 pandemic had changed “who you are as a person” and “your outlook on life”. However, both studies used younger, highly educated samples, and in one study [53] participants were mostly living in less disadvantaged areas, with 62% living in neighbourhoods with good, very good or excellent liveability based on factors related to housing, physical environment, amenities, residents’ demographics and safety. Our work therefore extends these findings, to show that the concept of the pandemic as a teachable moment for the importance of physical activity, also has some resonance among older adults living in areas of high socioeconomic deprivation.

We also found contrasting ideas in the way that physical activity was described by the older adults, with regards to whether it was something done for purpose or pleasure. A lot of the engagement in physical activity described by participants was often linked to purposeful tasks such as active transport, doing housework or caring for others. Only four participants reported that they were meeting current physical activity recommendations during leisure-time. This finding is in line with previous research showing lower levels of leisure-time physical activity amongst lower SES groups from North America, Europe and Australia [20, 22]. However, despite this a sense of enjoyment was also reported as an important factor to engaging in physical activity, and combining physical activity with other leisure interests was seen as a facilitator to engagement. This coincides with other research looking at the acceptability of engaging in physical activity amongst older adults residing in socioeconomically deprived areas in England, which found physical activity was more acceptable when it is enjoyable and addresses multiple needs including leisure interests [41]. The current study adds further insights to this, through finding that more traditional forms of physical activity such as formal exercise classes or going to the gym did not connect with this group and were often seen as lacking a sense of interest or enjoyment.

Many participants in the current study described how their physical activity had changed with age. This was expressed through either feeling too old to engage in certain types of physical activity or, more frequently, through reports of physical health limitations which were often cited as barriers to engaging in physical activity. This is supportive of other research looking at the acceptability of engaging in physical activity amongst older adults experiencing socioeconomic disadvantage in Australia, which found deteriorating health to be a barrier to engaging in physical activity, whilst modifying activities in accordance with individuals’ physical capabilities helped to facilitate engagement [39]. Many participants in the current study described adapting their levels of physical activity to suit their own physical capabilities.

With regards to opportunities to be physically active, the importance of having access to green space was reflected upon a lot in the current study, particularly during COVID-19 restrictions and its perceived importance has continued to endure post-pandemic. Outdoor physical activity was a key preference for many participants. This is in accordance with previous research from the Netherlands, which found that outdoor physical activities were seen as more pleasurable compared to those performed at home amongst older adults [55]. However, poor weather and neighbourhood safety issues were cited as barriers in the current study. Previous research has also highlighted neighbourhood safety as a barrier to engaging in physical activity, amongst older adults of lower SES in Northern Ireland [56]. A perhaps surprising finding of the current study, was that many of the participants reflected on having good access to facilities and activities, including leisure centres and organised physical activity classes and groups. This is in contrast with other research amongst older adults living in areas of higher socioeconomic deprivation in England, which found a perceived lack of availability of community activities [40], and a lack of services and loss of local facilities, leading to individuals feeling undervalued and disadvantaged [41]. However, some participants in the current study were not actively using facilities despite being aware of them, and some participants also reflected that spaces and activities need to be more age friendly to appeal to more older adults. One place which was unanimously unpopular amongst this group was gyms, due to them being indoors, having an overly serious atmosphere, a social pressure to keep up with other people, and limited opportunities for social connection. This coincides with other research amongst a highly active sample of older adults in the UK, which found that the traditional gym environment was not appealing, particularly for women, with participants citing a dislike of the atmosphere [57]. Whilst no details about the SES of the overall sample were given for this previous study, one participant did describe themselves as “middle class”. The findings from the current study extend on this work, by demonstrating that a dislike of a gym environment is also found in less active older adults living in socioeconomically deprived areas.

Time was often cited as a key barrier to engaging in more physical activity, due to competing commitments including caring, work, household chores and attending medical appointments. This is in accordance with previous research looking at the acceptability of engaging in physical activity interventions amongst older adults with lower SES [37]. An individual’s social environment was also an important factor with regards to the acceptability of engaging in physical activity amongst this group. There was a clear preference for group physical activity, which was seen as more fun than performing physical activity solo, and social connections were often a motivator for engaging in physical activity. This is well supported by other research amongst this population, which demonstrates that physical activity is more acceptable when it addresses needs of social connection [41]. Whilst social familiarity (i.e. being amongst peers with shared characteristics) helps facilitate engagement with physical activity [37]. Support and encouragement from family and friends was also seen as an important facilitator to engaging in physical activity. This is supported by evidence from a systematic review, with included studies from North America, Europe, Asia and Australia, which found that older adults with greater levels of social support for physical activity, were more likely to engage in leisure time physical activity, especially when this social support came from family members [58].

Most of the older adults in the current study were aware of how important physical activity is in later life, however there was a lack of understanding of how much physical activity is recommended for older adults. This finding resonates with numerous previous research studies, conducted pre-pandemic in the UK and USA, highlighting a lack of awareness and knowledge of physical activity guidelines amongst older adults [57, 59], and amongst lower SES groups [59–61]. Therefore, there is a clear and enduring need for more effective communication of physical activity guidelines amongst older adults from lower SES groups. In the current study, it was found that messaging and imagery which accompanied physical activity guidance felt unrelatable to participants. Participants also reflected how physical activity need not be complex. This is line with previous research conducted prior to the pandemic, looking at physical activity messaging preferences amongst older adults living in more deprived areas of the UK, which found a desire amongst this group for relatable imagery to enable people to believe physical activity is for them, in addition to simple messages free of jargon [62]. The findings from the current study, alongside those from previous research, demonstrate a clear and enduring need for more effective physical activity messaging and communication of guidelines amongst older adults from lower SES groups. Action is needed to develop messaging that is tailored and appropriate to the needs of this already vulnerable population. This is particularly important considering the increasing socioeconomic inequalities in physical activity levels since the onset of the pandemic [34, 36].

### Strengths and limitations

To our knowledge, this is the first study to explore the acceptability of engaging in physical activity after the COVID-19 pandemic, amongst older adults from lower SES groups. A strength of this study is that it adds to the understanding of engagement in physical activity, amongst this important target population for public health interventions to increase physical activity. This study also included a sample with gender parity and ethnic diversity, adding further insight into factors related to the acceptability of engaging in physical activity amongst older adults from lower SES groups. This builds on previous UK studies amongst this target population, which have tended to consist of mostly White and predominantly female samples [40, 41, 56].

However, a limitation of this study was that despite participants being recruited from areas of high socioeconomic deprivation, the sample was highly educated with just over 50% having achieved a Bachelor’s or Postgraduate degree. This may limit insight into the acceptability of engaging in physical activity amongst older adults with lower levels of education. Area-based and individual-level SES measures often diverge in how accurately they reflect socioeconomic disparities. For instance, recent research across Great Britain found that widely used area-level deprivation indices – such as the English, Scottish, and Welsh IMD —have low sensitivity and specificity. More than half of income- or employment-deprived individuals live outside the most deprived 20% of areas, meaning 55–62% of income-deprived and 56–63% of employment-deprived people are missed when using area-level identification alone [63]. Similarly, in the US, area-level proxies (e.g. census tract or county median income/education) moderately approximate individual SES, with census-tract measures outperforming county-level ones, but still substantially attenuate observed associations—especially with health outcomes like mortality—compared to direct individual-level measures [64]. Overall, while area-level SES can capture environmental or contextual factors impacting health, relying on them as proxies for individual-level disadvantage risks misclassification and may systematically underestimate socioeconomic inequalities. Practically, however, area-based measures are more useful when recruiting people. Further, policy in this space is often area-based and hence needs to target areas of high deprivation rather than specific individuals.

Another limitation of this study is that data was solely collected from individuals living in urban areas, and therefore this limits the insight into the experiences of older adults living in more rural areas of high socioeconomic deprivation. Many participants in this study felt they had good access to facilities and activities to participate in physical activity, including leisure centres and organised physical activity classes and groups. This may not be the case for those living in rural areas who may face different barriers, and have less opportunities available to them to engage in physical activity.

### Implications

This qualitative study offers important implications for the development of physical activity interventions amongst older adults from lower SES groups. These findings suggest that more effective physical activity messaging is needed, to better communicate physical activity guidelines amongst this population. Future research should work with older adults from lower SES groups to co-design messaging that is accessible and relatable to them. There is also a clear need to increase physical activity during leisure time amongst this population. Future research focused on physical activity messaging amongst this population, could look to emphasise messages of fun and enjoyment, to help market physical activity as something that can be done for pleasure rather than feeling like an obligation. This may help those who are insufficiently active to increase their intrinsic motivation to get active and be more inclined to make time for physical activity. Previous participatory research exploring physical activity messaging preferences, amongst community groups living in more deprived areas of the UK, found that amongst older adults within these communities, enjoyment and social connections were seen as highly valued benefits of engaging in physical activity, and as such were regarded as key content to include within physical activity messages [62]. However, the sample of older adults was small (*n* = 5), all were White British and 80% were female, so this limits insights. Future research should explore the preferences for physical activity messaging amongst a larger, more diverse sample to gain further insights. Useful starting points for future research to improve messaging, and interventions more broadly for this population, could be through promoting social benefits of physical activity, linking physical activity with other leisure interests, and incorporating different types of physical activity outside of the more formal exercise classes and gym-based activities. These may also be important factors for those involved with social prescribing of physical activity, who may wish to ensure referral schemes incorporate these factors when working with this population group. Findings from the current study demonstrate that the COVID-19 pandemic acted as a teachable moment for many with regards to physical activity, intervention development should seek to capitalise on this as individuals may be more receptive to change.

## Conclusions

Older adults from lower SES groups represent an important target population for interventions aimed at increasing physical activity. The present study provides in depth insight into the acceptability of engaging in physical activity in the aftermath of the COVID-19 pandemic, amongst older adults living in areas of high socioeconomic deprivation in Manchester, England. The COVID-19 pandemic was seen as a teachable moment with regards to physical activity, demonstrating that now is a good time to act for intervention development, to capitalise on the increased motivation to do more physical activity amongst this population. Engagement in physical activity during leisure time is limited amongst this population, despite this a sense of enjoyment is important to the acceptability of physical activity. To increase engagement in leisure-time physical activity future interventions should look to emphasise fun. Fostering opportunities for social connection, providing safe opportunities to engage in outdoor physical activities, linking physical activity with other leisure interests, and moving away from more formal exercise classes and gym-based activities, which are unappealing to this population, may be solutions to provide this. Physical health limitations, internalised ageism and limited time are barriers to engaging in more physical activity amongst this group, whilst support from family and friends is a facilitator. There is a sense of the importance of physical activity in older age, but a lack of knowledge with regards to how much physical activity is recommended for older adults. Better messaging is needed to communicate and improve the accessibility of physical activity guidelines amongst this group. Future messaging could look to emphasise messages of fun and enjoyment to help market physical activity as something that can be done for pleasure.

## Supplementary Information

Supplementary Material 1.

Supplementary Material 2.

## Data Availability

The dataset supporting the conclusions of this article is available in the Figshare repository, [10.48420/29987035.v1](10.48420/29987035.v1).

## Abbreviations

CVD: Cardiovascular disease
IDAOPI: Income Deprivation Affecting Older People Index
IMD: Index of Multiple Deprivation
PASE: Physical Activity Scale for the Elderly
SES: Socioeconomic status
WHO: World Health Organization
