# Supplementary material for: Exploring the acceptability of engaging in physical activity amongst older adults living in socioeconomically deprived areas after the COVID-19 pandemic: a qualitative study
Source: BMC Public Health. 2025 Oct 29;25:3664. doi: 10.1186/s12889-025-24981-6 (PMC12574018; doi:10.1186/s12889-025-24981-6)
Supplement: Supplementary file 1 — Supplementary Material 1. [file 12889_2025_24981_MOESM1_ESM.docx]

**Interview Topic Guide**

**Opening statement:** Thank you for agreeing to participate in this interview. I’ll be asking you some questions about your experiences of and feelings towards physical activity during and since the COVID-19 pandemic. When talking about physical activity this can include physical activities you do during your leisure time, around the house and garden or at work. There are no right or wrong answers. I just want to hear your views. You can take a break at any time, and you don’t have to answer a question if you do not want to. Does that sound okay?

1. **What do you understand by the term ‘physical activity’?**

- How much do you know about any recommendations/guidelines about physical activity for older adults?
- If aware of guidelines:
- How important do you feel these guidelines are to you?
- Do you attempt to follow those guidelines?
- How easy do you think it is for older people to meet these guidelines?

1. **How would you describe yourself in terms of physical activity levels?**

- If active – What motivates you to be so active?
- If not – What stops you from being more active? What would need to change for you to increase your level of physical activity?

1. **How do you feel about physical activity?**

- If enjoy - What kinds of physical activity do (did) you enjoy/enjoy the most? (If references made to previous enjoyment but not present enjoyment, find out what has changed to stop them enjoying it now and what could help them to enjoy it again).
- If don’t enjoy PA - What is it about physical activity that you don’t like?
- How important is physical activity to you?
- What benefits do you think being physically active has for you? OR What benefits do you think there would be from being more physically active? (Explore physical, psychological and social benefits)
- Is there anything that would worry you about increasing your level of physical activity? What would ease those worries?

1. **What are the ranges of activities available, like in your area? Are there any physical activities that you would like to do, but do not have access to?**

- How would you prefer it to be delivered? Time of day? Number of times per week? Location?

1. **What makes it easier/harder for you personally to be physically active?**
2. **What influence do others have on how physically active you are?**

- Do they provide any encouragement? Do they join in as well?

1. **Has any health professional ever suggested that you become more physically active?**

- Yes - How did you feel about that? What was the effect? What could they have done differently?
- No - How would you feel if one did?

1. **How have your activity levels changed if at all since the pandemic compared to before the pandemic?**

- If less active – What has stopped you from being as active?
- If more active – What has motivated you to be more active?

1. **How has the pandemic changed if it all the way you feel about doing physical activity?**

- Explore levels of confidence in doing physical activity
- Explore feelings of importance of doing physical activity

1. **How has the range of activities in your local area changed if at all since the pandemic?**

- Explore availability of face-to-face vs. digital activities
- Reduced days of the week open? Reduced hours opened?

1. **How has the pandemic changed if at all how easy or difficult it is for you to be physically active?**

- Do you feel as able to do physical activity as you were before the pandemic?

1. **How do you feel about adding in more physical activity into your day-to-day routine *today?**

- Is this something you would be comfortable to attempt?
- How easy or difficult do you think you would find this?
- What kind of problems or barriers do you think you might encounter in trying to do this? What might help you to overcome these?
- How confident are you that you would be successful in attempting to increase your physical activity? How confident that changes could be maintained? What would help you to maintain changes?

1. **Are there any other important issues or anything you would like to add that we haven’t discussed?**
